# Role of HOXA7 to HOXA13 and PBX1 genes in various forms of MRKH syndrome (congenital absence of uterus and vagina)

**DOI:** 10.1186/1477-5751-5-4

**Published:** 2006-03-23

**Authors:** Agnès Burel, Thomas Mouchel, Sylvie Odent, Filiz Tiker, Bertrand Knebelmann, Isabelle Pellerin, Daniel Guerrier

**Affiliations:** 1 CNRS UMR 6061, Génétique et Développement, Université de Rennes 1, Groupe IPD, IFR140 GFAS, Faculté de Médecine, Rennes, France; 2 Service de Gynécologie Obstétrique, CHU de Rennes, Rennes, France; 3 Unité de Génétique Médicale, Hôpital Sud, Rennes, France; 4 Department of Pediatrics, Baskent University, Adana Hospital, Adana, Turkey; 5 Service de Néphrologie, Hôpital Necker-Enfants-Malades, Paris, France

## Abstract

The Mayer-Rokitansky-Küster-Hauser (MRKH) syndrome refers to the congenital absence or severe hypoplasia of the female genital tract, often described as uterovaginal aplasia which is the prime feature of the syndrome. It is the second cause of primary amenorrhea after gonadal dysgenesis and occurs in ~1 in 4500 women. Aetiology of this syndrome remains poorly understood. Frequent association of other malformations with the MRKH syndrome, involving kidneys, skeleton and ears, suggests the involvement of major developmental genes such as those of the HOX family. Indeed mammalian HOX genes are well known for their crucial role during embryogenesis, particularly in axial skeleton, hindbrain and limb development. More recently, their involvement in organogenesis has been demonstrated notably during urogenital differentiation. Although null mutations of HOX genes in animal models do not lead to MRKH-like phenotypes, dominant mutations in their coding sequences or aberrant expression due to mutated regulatory regions could well account for it. Sequence analysis of coding regions of HOX candidate genes and of PBX1, a likely HOX cofactor during Müllerian duct differentiation and kidney morphogenesis, did not reveal any mutation in patients showing various forms of MRKH syndrome. This tends to show that HOX genes are not involved in MRKH syndrome. However it does not exclude that other mechanisms leading to HOX dysfunction may account for the syndrome.

## Background

The most common cause of vaginal agenesis is congenital absence of the uterus and vagina which is also referred to as Müllerian aplasia, Müllerian agenesis or Mayer-Rokitansky-Küster-Hauser (MRKH) syndrome [1]. The frequency of this syndrome is not yet entirely clear, although reported incidences vary from 1 in 4,000 to 5,000 female births [1-3]. Affected individuals are clearly phenotypic females with normally developed ovaries [4,5] and normal 46, XX karyotype [6,7]. Aetiology of the syndrome is poorly understood but it is often associated with other anomalies including renal defects, skeletal abnormalities and deafness (MURCS association [8]), suggesting the involvement of major developmental genes such as HOX genes [9-12].

The homeobox (HOX) genes belong to a large family of 39 genes organized in four clusters, HOXA, HOXB, HOXC and HOXD, each on a different chromosome. During organogenesis, the proteins encoded by these genes act through various and highly complex spatiotemporal combinations to trigger positional identity of embryonic cells. This determines the patterning and segment identity along the anterior-posterior axis of the skeleton and a variety of organ systems [13]. For instance, 30 HOX proteins participate to the elaboration of the spine, 12 for the digestive tract and 7 for the urogenital tract [14]. More precisely, Müllerian ducts (the primordia for oviducts, uterus, cervix and anterior vagina) development seems to involve relatively few HOX genes in the mouse model. Indeed, HOXA7 [15], HOXA9 to HOXA13 [16], as well as HOXD9 to HOXD13 [17], are expressed along the differentiating Müllerian duct. However, alteration of the female genital tract is only observed in HOXA10, -A11 and -A13 deficient mice (homozygotic inactivation of the gene): – in HOXA10 -/- mice, the upper part of the uterus is transformed into oviduct, the uterotubular junction is abnormal as well as the uterine epithelium and an anterior homeotic transformation of lumbar vertebrae has occurred [18]; – in HOXA11 -/- mice, the uterus is thinner and shorter than normal and endometrial glands have not developed [19]; in HOXA13 -/- mice, the distal Müllerian duct has not developed [20]. Finally HOXA10 to HOXA13 are also expressed in the developing kidney [21] and are both required for correct patterning of the skeleton [22].

HOX proteins share in common a highly conserved 60 amino acid DNA binding motif referred to as the homeodomain. Proteins containing this domain are regulatory factors that control expression of target genes [23]. Their high biological specificity comes from cooperation with specific cofactors that contribute to modulate DNA binding specificity. Members of the three amino-acid loop extension (TALE) class of homeodomain proteins that comprise the mammalian PBX proteins [24] and the MEIS-like TALE factors or MEINOX group (mammalian MEIS and PREP1 proteins) [25] are now considered as essential cofactors forming heterotrimeric complexes with HOX proteins that regulate specific target gene transcription [26]. Among these cofactors, PBX1 is of great interest in regards to malformations found in MRKH syndrome: it is required for skeletal development and patterning [27], kidney morphogenesis [28] and especially, its gene inactivation leads to absence of Müllerian structures [29]. Interestingly, PBX1 is expressed in the Müllerian ducts at the onset of genital tract differentiation whereas it is absent of Wolffian ducts (the primordia for male inner genital tract) during the same period and in both sexes [30].

These overall data led us to investigate HOXA7, -A9, -A10, -A11 and -A13 genes, as well as PBX1, in several MRKH patients showing a wide range of malformations, from isolated uterovaginal aplasia to severe MURCS association. However null mutations of these genes do not result in MRKH-like phenotype in the mouse model. This is why we decided to search for simple or discrete mutations within their coding and splicing sequences. Indeed, dominant or loss-of-function mutations can impair ability of the corresponding proteins to fulfil their biological role as already showed for HOXD13 [31,32].

## Case reports

### Patient 1

This patient was initially evaluated for a vesicoureteric reflux that required surgical treatment during which a small left kidney and a partial uterine agenesis with rudimentary left horn were noticed. This was confirmed latter by laparoscopy when she was 13 year old. Additional examination revealed several skeletal abnormalities: coxa valga, unequal leg length, flexus adductus as well as L4 vertebra and sacrum malformation. At 18 year of age, laparoscopic-assisted Vechietti procedure [33] was performed. Finally her karyotype was normal.

### Patient 2

This 25-year-old white woman was initially evaluated for proteinuria. Examination revealed a right single pelvic kidney and uterovaginal agenesis. She had normal sexual secondary development. Kidney biopsy showed focal and segmental hyalinosis. Spine radiograms were normal. Her karyotype was normal. At 26, she was treated by sigmoid colpoplasty [34]. During surgery, uterovaginal agenesis was confirmed with small rudimentary uterine horns.

### Patient 3

This 20-year-old white woman was evaluated for primary amenorrhea. She had normal secondary sexual development. There was no cyclic abdominal pain. Family history was unremarkable. The MRKH diagnosis was confirmed by laparoscopy. Absence of right ovary and fallopian tube was noticed during surgery. However, ultrasound examination showed normal kidneys.

### Patients 4 to 6

These patients are three Turkish sisters already described [35] (patients III2, III3, III5 of pedigree). Interestingly, in this family, the fourth sister (III4) was not affected but two paternal aunts (II6 and II7), among 8 siblings, were sterile and were told they had no uterus. This three sisters case corresponds to typical MRKH syndrome with primary amenorrhea, normal sexual secondary development and absence of the vagina at physical evaluation. The Müllerian agenesis was confirmed by ultrasound examination and magnetic resonance imaging of pelvis. Their karyotypes were normal. Intravenous pyelogram and spine radiograms were normal in each case.

## PCR Amplification and sequencing

Total genomic DNA was prepared from peripheral blood leukocytes according to standard procedures [36]. Local ethical review and consenting procedures were followed. PCR primers were designed to amplify HOXA7, HOXA9, HOXA10, HOXA11, HOXA13 (Table 1) and PBX1 coding exons (Table 2). PCR reactions were carried out in 25 μl containing 500 ng genomic DNA, PCR buffer (50 mM KCl, 10 mM Tris HCl, pH 9.0), 1.5 mM MgCl2, 0.2 mM dNTP, 10 pmol of each primer, and 2.5 U *Taq *polymerase (Promega). PCR amplification was carried out using the "touchdown" methodology, with an initial denaturation step at 96°C for 3 min. followed by 19 touchdown cycles of 45 s at 96°C, 45 s at an initial melting temperature (Tm) of 69°C (with a 1°C Tm decrease by each cycle), and 60 s at 72°C. Amplification was then achieved by 11 cycles of 45 s at 96°C, 45 s at 50°C, and 60 s at 72°C, with a final extension at 72°C for 10 min. For the N-terminal exon 1 of HOXA13 gene, DMSO (5%) was added to PCR mix. 6 μl PCR product previously controlled on a 2% agarose gel, was incubated with 5 units of exonuclease I (Amersham Biosciences) and 1 unit of shrimp alkaline phosphatase (Amersham Pharmacia) in order to digest remaining primers and to inactivate unincorporated nucleotides. The enzymatic reaction was stopped by a step at 90°C for 15 min. Bidirectional sequencing of the PCR products was achieved using the BigDye Terminator chemistry (PE Applied Biosystems) and each of exon-specific primers. Electrophoresis and analysis were performed on an ABI Prism 377 (PE Applied Biosystems). Sequences were analyzed and compared with sequences downloaded from GenBank by DNAStar software (DNAStar).

**Table 1:** Forward (F) and reverse (R) primers used for PCR-mediated amplification of genomic DNA of HOXA7 to HOXA13 genes exons.

**Primer name**	**Gene segment**	**Sequence 5'-3'**	**Product size (bp)**
HOXA 7-1-F HOXA 7-1-R	HOXA-7 exon 1	TTGGTGTAAATCTGGGGGTG TTAAAACCAGAAAGGCTGCG	637
HOXA 7-2-F HOXA 7-2-R	HOXA-7 exon 2	GACTAGGCCAGGAGGAAGGT GGGAGCTGGAGTAGGTGATG	697
HOXA 9-1a-F HOXA 9-1a-R	HOXA-9 exon 1 (first half)	TGCCACCAAGTTGTTACATGA CAGCGGTTCAGGTTTAATGC	492
HOXA 9-1b-F HOXA 9-1b-R	HOXA-9 exon 1 (second half)	GCAGGTACATGCGCTCCT AAGGCAGGCTCGAGAGAAAC	356
HOXA 9-2-F HOXA 9-2-R	HOXA-9 exon 2	TGTGCGTCTTCTGCTCCTAA CGGACAGTTCTTTCTTTTTCTCTC	343
HOXA 10-1a-F HOXA 10-1a-R	HOXA-10 exon 1 (first half)	CTCCTGGCCCATCAATACAG GAGACTTTGGGGCATTTGTC	728
HOXA 10-1b-F HOXA 10-1b-R	HOXA-10 exon 1 (second half)	GCGCAGAACATCAAAGAAGA TCCTTGTGTCTGCCTGTCTG	535
HOXA 10-2-F HOXA 10-2-R	HOXA-10 exon 2	TGGCCTCGACTTAATCATCC AGACAGAGGGAGGGGACCAG	378
HOXA 11-1a-F HOXA 11-1a-R	HOXA-11 exon 1 (first half)	CAGCTGCAGTGGAGAATCAT CTTCTCGGCGCTCTTGTC	562
HOXA 11-1b-F HOXA 11-1b-R	HOXA-11 exon 1 (second half)	TTTTTCGAGACAGCCTACGG TGCGCTAGATTTCCAACTCC	340
HOXA 11-2-F HOXA 11-2-R	HOXA-11 exon 2	CTCACCCCATGCCTTTTCT GTCAAGGGCAAAATCTGCAT	331
HOXA 13-1a-F HOXA 13-1a-R	HOXA-13 exon 1 (first half)	ACTGGGGTCTTCTCCATGC TGGTGGTAGAAGGCGAACTC	727
HOXA 13-1b-F HOXA 13-1b-R	HOXA-13 exon 1 (second half)	CAACGCCATCAAGTCGTG AAGACCAGGGCTGGGAATAG	389
HOXA 13-2-F HOXA 13-2-R	HOXA-13 exon 2	CCGATCCCTGTGTAACTTGC ATTATCTGGGCAAAGCAACG	331

**Table 2:** Forward (F) and reverse (R) primers used for PCR-mediated amplification of genomic DNA of PBX1 gene exons.

**Primer name**	**Gene segment**	**Sequence 5'-3'**	**Product size (bp)**
PBX1-1-F PBX1-1-R	PBX1 exon 1	TTTCCCCCTTCCCTGTTTAT GTGATTCGGTTCCCATTGTT	334
PBX1-2-F PBX1-2-R	PBX1 exon 2	CAAATGTTTTCACCCTGTGC TTTGTGACTGCTGGTTAAGTGA	223
PBX1-3-F PBX1-3-R	PBX1 exon 3	TGGCAGCTTATGTAGCCAAA GTTGTGCTTCCTCCACCCT	404
PBX1-4-F PBX1-4-R	PBX1 exon 4	GCCCACGTGGCCTAATGTCATA TGGGGTGAAACTAGAGCCTG	372
PBX1-5-F PBX1-5-R	PBX1 exon 5	TGCTCCAAATTCACCTTTTG AAGACCTCTAAGAGCCTGCC	331
PBX1-6-F PBX1-6-R	PBX1 exon 6	TTCACCTCTCCCATAAAGCC CCCAATGTAGGAACAGCCAG	324
PBX1-7-F PBX1-7-R	PBX1 exon 7	GGTTGCTTTGCATGTCATTC TCTTGATTTTGGTTCGGTCG	354
PBX1-8-F PBX1-8-R	PBX1 exon 8	TCTGCCTCCCTTTTCCTACA GATGGCATGACCGATACAGA	304
PBX1-9-F PBX1-9-R	PBX1 exon 9	AAACAGCCACCCAATCTCAG TGTTTGCTGATTGCTTCGAC	261

## Results and discussion

The pattern of malformations observed in MRKH patients was, in our hypothesis, in favour of a HOX gene dysfunction. However no mutation as well as length/nucleotide polymorphism was found in the coding sequences of HOXA7 to -A13 genes of the patients we investigated. This probably refutes the hypothesis of dominant or loss-of-function mutations like those found in HOXD13 [31,32] and seems to show that quality of the corresponding proteins, if correctly expressed, can not be incriminated. Interestingly, reduced quantity of HOXA proteins (haploinsufficiency of the entire HOXA gene cluster) does not cause any of the major malformations observed in MRKH syndrome but leads to other congenital anomalies [37]. Nevertheless, other mechanisms can be suggested, such as upstream misregulation of some genes of the HOXA cluster, post-transcriptional anomalies, HOX partners' deficiency or defaults in HOX-target genes, all potentially leading to HOX-like phenotypes.

HOX genes clusters undergo very complex transcriptional controls during development, including general switch such as retinoic acid induction [38], FGFs [39,40] or Wnt [41] signalling, self-regulatory loops, specific induction or repression of HOX genes within the same cluster [42-44], as well as post-transcriptional regulations [45,46]. Although large-scale developmental signals deficiency would probably not account for restricted and non lethal malformations such as those observed for the MRKH syndrome, HOX misregulation due to mutations/deletions outside the coding regions could do it as already described in the HOXD gene cluster [47] and in HOXA13 gene promoter [48]. Some few regulatory regions have been characterized in the HOXA gene cluster among which, the so-called HCR (Human Control Region) [49] lying next to HOXA7, a gene somehow involved in Müllerian differentiation [15]. This 1.1 kb DNA sequence, as well as its conserved mouse equivalent, has been shown to set the anterior boundary of HOXA7 expression [49] and therefore putative other HOXA genes of the same cluster. Southern-blot experiments aiming at detecting length polymorphism such as deletion or duplication in the [HCR-HOXA7] area did not reveal any major genetic event in any of the patients investigated (results not shown). This however does not imply that other regulatory regions still uncharacterized in the HOXA cluster, may not be involved in the MRKH syndrome.

Post-transcriptional regulations also take place in the overall mechanisms of HOX gene expression and participate to the elaboration of the code referred to as "combinatorial HOX code". In this way, normal and alternative splicing of HOX pre-messengers [45,46] often results in two isoforms that putatively can antagonize each other [50,51]. In our experimental approach, we designed PCR/sequencing primers so that we were able to verify the correct splicing acceptor and donor sites sequences of all exons for every gene investigated (including PBX1). No mutation was found in these sites.

PBX1 is one of the HOX genes' partners the most likely to be involved in the MRKH syndrome. Heterozygotic (+/-) inactivation of this gene does not provoke any congenital malformation in the mouse model whereas homozygotic (-/-) mice embryos die before birth due to multiple and severe malformations [27]. Therefore haploinsufficiency will probably not cause MRKH phenotype although mono-allelic mutations in a coding region of the gene may well lead to a dominant and deleterious effect such as titrating of HOX proteins clustered in non functional complexes. We carefully sequenced the overall exons of PBX1 in every patient and did not observe any mutation.

## Conclusion

Investigation of candidate genes in biomedical research has often been unsuccessful unless target genes were obvious (for instance, see [52-54]). HOX genes, which play numerous roles during development, were good candidates for MRKH syndrome, based on deduction from their expression pattern during mouse development and from the phenotype of mice with a targeted disruption or overexpression of a specific HOX gene. Similar hypotheses were assumed for others congenital malformations or syndromes and revealed the involvement of these genes [55,56]. We based the present work on the investigation of MRKH patients showing various malformations associated with uterovaginal aplasia. This choice was based on the probable multigenic origins of the syndrome, assuming that at least one case would lead to evidence mutation of either a coding sequence of a HOX gene or part of the HOXA cluster (HOXA7 to -A13). Amongst the various MRKH cases analysed, we did not find any mutation in the coding sequences or in the [HCR-HOXA7] region. However, we did not sequence the whole HOXA cluster in every patient as this would have been a tremendous work but rather targeted genomic regions (coding sequences, splicing sites, regulatory sequences). Our negative results therefore do not mean that HOX genes are not involved in the syndrome. Additional investigation is necessary to settle or not the HOX hypothesis. This requires performing genetic linkage analysis of familial cases and whole-genome scan to seek for candidate chromosomal loci.

## Authors' contributions

- AB was in charge of most of the PCR and sequencing reactions

- TM co-initiated this program and delineated MRKH syndromes in patients 1 and 3

- SO contributed to the diagnosis and was in charge of medical genetics

- FT provided biological samples of patients 4–6

- BK provided biological samples of patient 2

- IP created a new research group focused on molecular events triggering normal and pathological differentiation of the Müllerian ducts. She therefore offered the opportunity to DG to set up a proper clinical research program aiming at understanding the genetics of MRKH syndrome.

- DG initiated the study in IP's group and has been leading this research program since then.
